# Updated canine infection rates for *Dirofilaria immitis* in areas of Brazil previously identified as having a high incidence of heartworm-infected dogs

**DOI:** 10.1186/s13071-014-0493-7

**Published:** 2014-11-07

**Authors:** Norma Vollmer Labarthe, Jonimar Pereira Paiva, Larissa Reifur, Flavya Mendes-de-Almeida, Alexandre Merlo, Carlos Jose Carvalho Pinto, Paulo Sérgio Juliani, Maria Angela Ornelas de Almeida, Leucio Câmara Alves

**Affiliations:** Programa de Pós-Graduação em Medicina Veterinária, Faculdade de Veterinária, Universidade Federal Fluminense, Rua Vital Brazil Filho 64, Santa Rosa, CEP 24230-340 Niterói, RJ Brazil; Programa Institucional Biodiversidade e Saúde, Fundação Oswaldo Cruz, Av. Brasil 4036, Manguinhos, CEP 21040-361 Rio de Janeiro, RJ Brazil; Universidade Federal Rural do Rio de Janeiro, Seropédica, RJ Brazil; Programa de Pós-Graduação em Microbiologia, Parasitologia e Patologia, Departamento de Patologia Básica, Universidade Federal do Paraná, Av. Cel. Francisco H. dos Santos, 100, CEP 81530-900 Curitiba, PR Brazil; Zoetis, Rua Alexandre Dumas, 1711, 8th floor, tower B, CEP: 04717-000 São Paulo, SP Brazil; Departamento MIP/CCB/UFSC, Campus Universitário, Trindade, CEP 88040-900 Florianópolis, SC Brazil; Universidade de São Paulo, Via Santos Dumont 405, Jardim Santo Antônio, CEP: 11432-501 Guarujá, SP Brazil; Escola de Medicina Veterinária e Zootecnia da Universidade Federal da Bahia, Av. Ademar de Barros, 500, Ondina, CEP 40170-110 Salvador, BA Brazil; Departamento de Medicina Veterinária da Universidade Federal Rural de Pernambuco, Av. Dom Manoel de Medeiros s/n, Dois Irmãos, CEP 52171-900 Recife, PE Brazil

**Keywords:** Canine antigen tests, Canine heartworm, Clinical signs, *Dirofilaria immitis*

## Abstract

**Background:**

Canine heartworm infections were frequently diagnosed in Brazil before the new millennium. After the year 2000, the frequency of diagnosis showed a sharp decline; however, a few years later, new evidence indicated that the parasite was still present and that canine infection rates seemed to be increasing. Therefore, an updated survey of canine heartworm prevalence was conducted in several locations in south, southeast, and northeast Brazil.

**Methods:**

Dogs from 15 locations having previously reported a high prevalence of heartworm infection were included in the survey according to defined criteria, including the absence of treatment with a macrocyclic lactone for at least 1 year. Blood samples from 1531 dogs were evaluated by an in-clinic immunochromatography test kit (Witness® Heartworm, Zoetis, USA) for detection of *Dirofilaria immitis* antigen. At each location, epidemiologic data, including physical characteristics and clinical signs reported by owners or observed by veterinarians, were recorded on prepared forms for tabulation of results by location, clinical signs, and physical characteristics.

**Results:**

The overall prevalence of canine heartworm infection was 23.1%, with evidence of heartworm-infected dogs detected in all 15 locations studied. There was a tendency for higher prevalence rates in environmentally protected areas, despite some locations having less-than-ideal environmental temperatures for survival of vector mosquitoes. Among physical characteristics, it was noted that dogs with predominantly white hair coats and residing in areas with a high (≥20%) prevalence of heartworm were less likely to have heartworm infection detected by a commercial heartworm antigen test kit than were dogs with other coat colors. In general, dogs older than 2 years were more frequently positive for *D. immitis* antigen than were younger dogs. Clinical signs of heartworm infections were rare or owners were unable to detect them, and could not be used for reliable prediction of the presence of heartworm.

**Conclusions:**

These results indicate that the prevalence of *D. immitis* has increased in these areas of Brazil over the past few years. Small animal practitioners in these areas should include routine screening tests for heartworm infections in every dog’s annual evaluation protocol and make sure to have uninfected dogs on prevention.

## Background

*Dirofilaria immitis* (Leidy, 1856) Raillet & Henry, 1911, is a mosquito-borne parasite species distributed throughout all continents although in different prevalences. The prevalence of the parasite in Brazil was reported to be 8% during the 1980s [1], with hotspots that could be as high as 45% in the coastal lowlands of the state of São Paulo [2] and in the eastern lowland section of the state of Rio de Janeiro [3]. Several canine heartworm hotspots were identified in coastal areas such as Florianópolis (12%), state of Santa Catarina [4]; Guaratuba (6%) and Guaraqueçaba (21%), state of Paraná [5]; Bertioga (45%) and Guarujá (14%), state of São Paulo [2], região dos lagos (52%) and Niterói (37%), state of Rio de Janeiro [3]; Recife (12%) [6] and Itamaracá (29%) [7], state of Pernambuco; and Salvador (5.4%) and Lauro de Freitas (23.3%), state of Bahia [8]. As awareness of the disease increased following introduction of chemoprophylatic drugs in Brazil and with the increased treatment of canine tick-borne diseases, the number of heartworm-infected dogs declined. At the beginning of the new millennium (2001), the reported national prevalence of heartworm infection in Brazil was 2%, while *Ehrlichia canis* seroprevalence was 30% [9]. The reasons for the downward trend in *D. immitis* infection in Brazil included appropriate use of chemoprophylaxis, widespread use of off-label injectable ivermectin, and increased use of tetracycline (or derivatives) to control ehrlichiosis [10] that may affect the *Wolbachia* endosymbiont as well as the survival and reproduction of adult heartworm [11].

Following the observed decline in the prevalence of heartworm in the early years of the new millennium, the first report of a Brazilian outbreak was in the state of Rio de Janeiro, at the eastern lowland section [12]. Subsequent to this report, small animal practitioners from different areas of the country began to detect heartworm infections in dogs in their clinics during routine blood work [13]. Despite the apparent increase in reports of infected dogs, there are no updated systematic surveys conducted in Brazil. Therefore, the need for an update on canine heartworm prevalence in Brazil is unquestionable.

The damage promoted over time to pulmonary arteries, right ventricle, and to all vascular structures near the lungs by the adult worms often leads to severe disease [14], generally recognized by clinical signs, such as coughing, dyspnea, and exercise intolerance [15,16]. Although the disease is well known in the literature, many times it is difficult to be identified by owners and veterinarians; thus, infected dogs may not receive prompt treatment for heartworm infection. Therefore, there is an urgent need to convince small animal practitioners to include heartworm testing as a routine examination for their dogs even when owners report the absence of any clinical signs that could suggest *D. immitis* infection.

Considering that updated data regarding the prevalence of *D. immitis* in areas of Brazil is warranted and that reliable clinical signs of heartworm infection in dogs are missed or underestimated by owners, the present article reports the prevalence of canine heartworm infection at different sites in the coastal area of Brazil as well as dog-owners’ perceptions regarding clinical signs of the presence of heartworm infection. These data are expected to aid veterinary practitioners for better and earlier diagnosis of canine heartworm infection in their practice.

## Methods

The study was designed to include approximately 1600 dogs from areas previously identified as having high rates of heartworm infection. Dogs were selected for inclusion in the study according to the following criteria: i) dogs should have lived at the location for at least 1 year; ii) if possible, there should be no more than three dogs kept in the home; iii) dogs could have not received any treatments with macrocyclic lactones for at least 12 months; and iv) a formal consent must be signed by owners. The protocol was approved by the committee of animal use (CEUA) of the Universidade Federal Rural do Rio de Janeiro.

**Figure 1:**
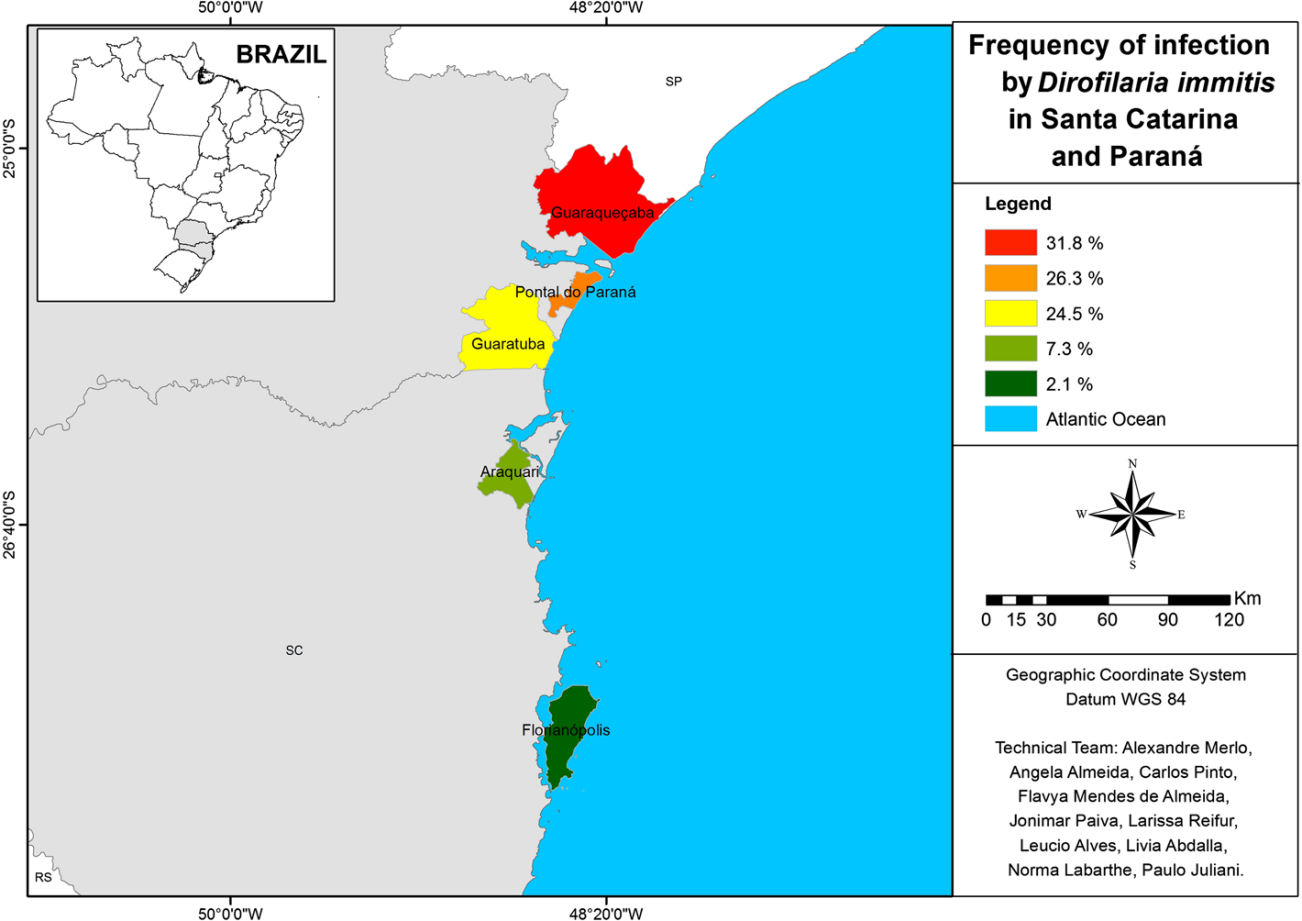
**Map showing the percentage of canine positive test results for heartworm antigen in southern Brazil.**

**Figure 2:**
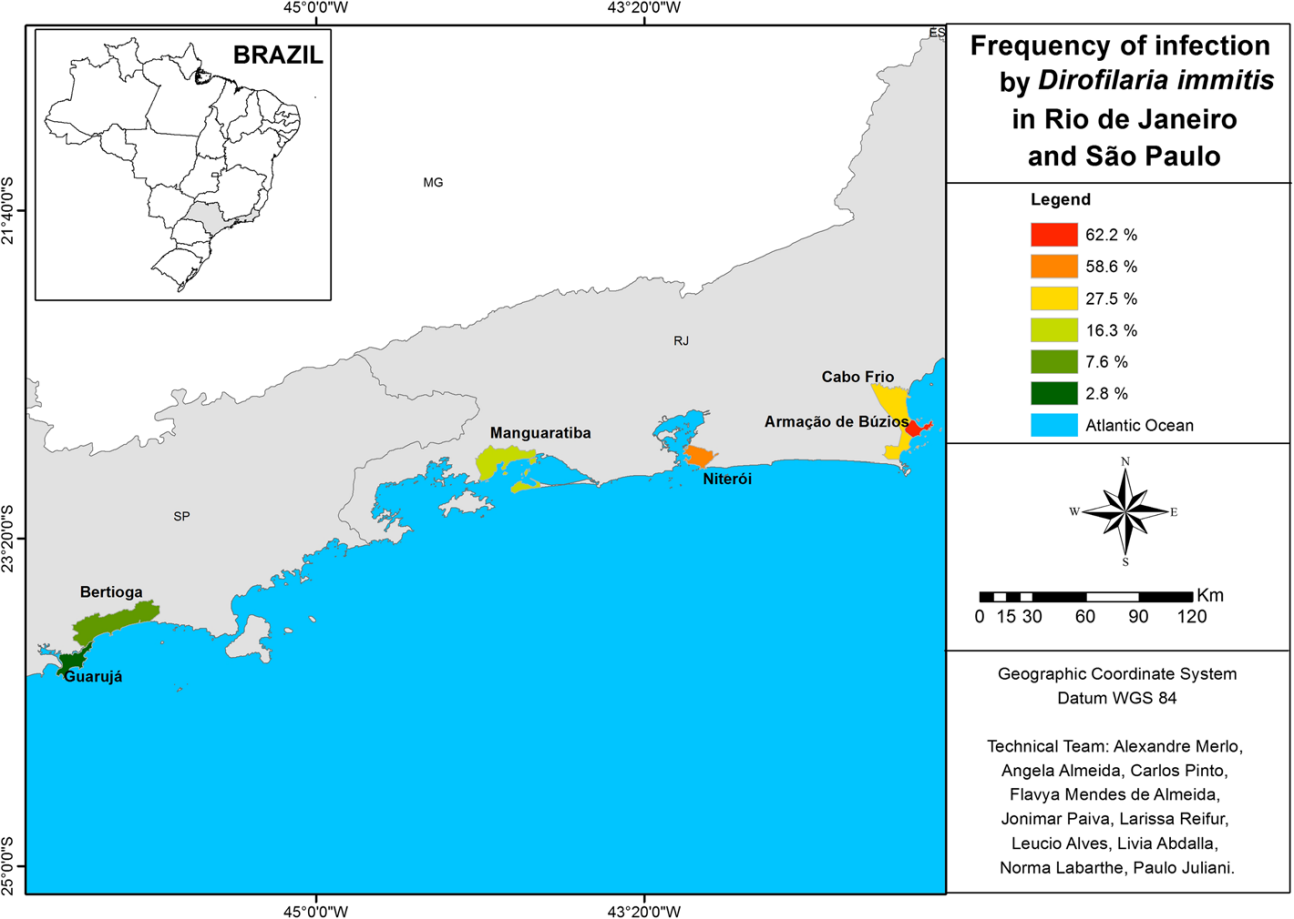
**Map showing the percentage of canine positive test results for heartworm antigen in southeastern Brazil.**

**Figure 3:**
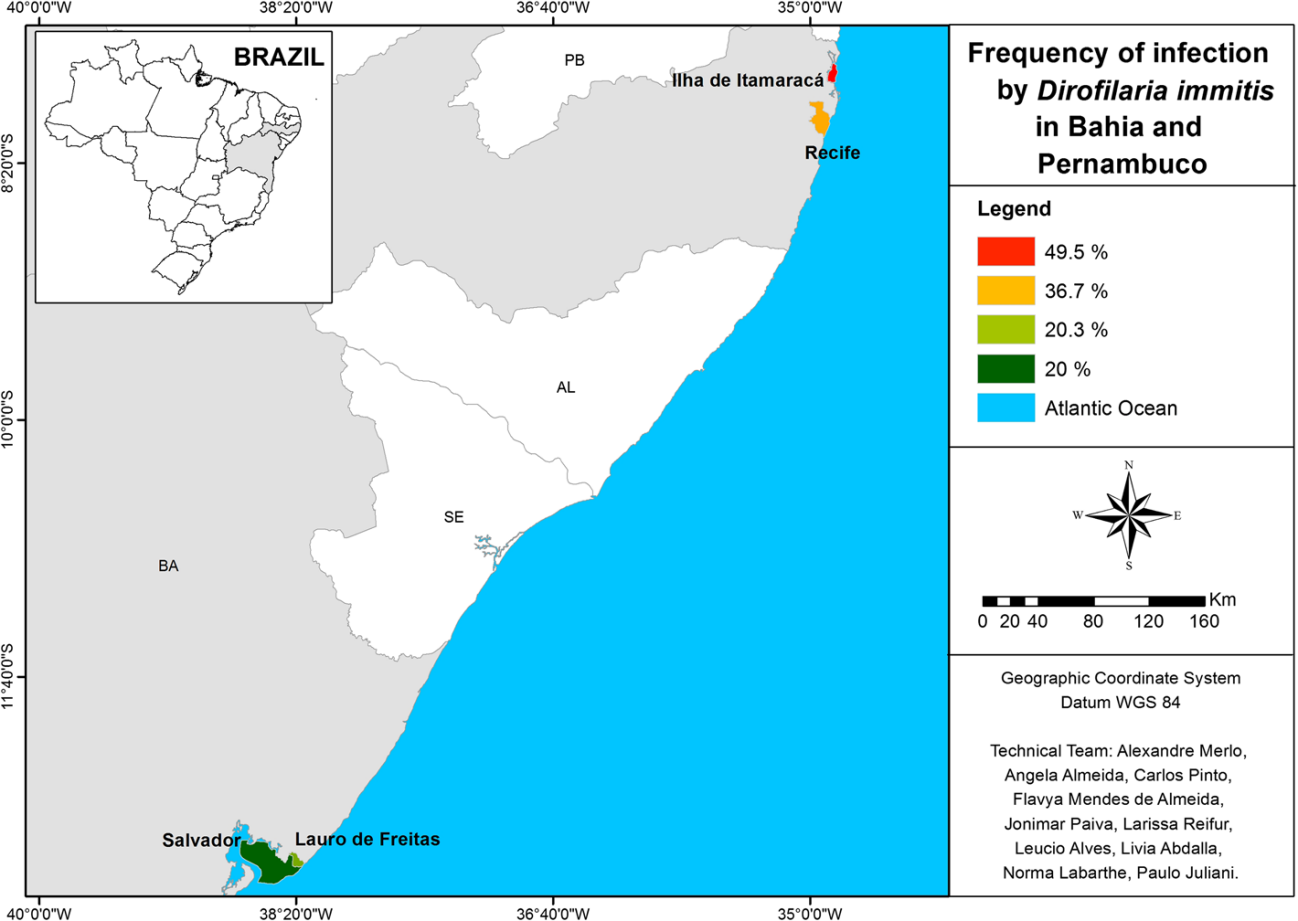
**Map showing the percentage of canine positive test results for heartworm antigen in northeastern Brazil.**

Along the Brazilian coast, the states Santa Catarina, Paraná, São Paulo, Rio de Janeiro, Bahia, and Pernambuco were included in the survey (Figures 1, 2 and 3). These states had previously been reported to have high rates of heartworm infection. The minimum number of samples to be obtained in each state was calculated with Epi Info 2000 (Centers for Disease Control and Prevention, Atlanta, Georgia) for determining 90% confidence intervals, considering the canine population to be 15% of the human population [17] and the estimated heartworm prevalence to be as reported previously [2,4-8].

Blood samples were obtained from dogs presented for rabies vaccination or spay and neuter campaigns. At places where no such campaigns were available, active search for homes with dogs was used, avoiding buildings that were multiple-housing units. At each locale, epidemiologic data, including each dog’s characteristics and clinical signs, were recorded on prepared survey forms. Blood samples were processed to obtain plasma to be tested by an in-clinic immunochromatography test kit (Witness® HW (heartworm) antigen test kit, Zoetis, USA) for detection of *D. immitis* antigen.

Test results were compiled by locale, clinical signs, and dog characteristics for determination of statistical significance by chi square or Fisher exact tests.

## Results

A total of 1531 blood samples were obtained from September 2013 through March 2014, reaching the minimum sample size for a 90% confidence interval. Overall, 23.1% of the samples examined were positive for heartworm antigen (Table 1). Heartworm-infected dogs were diagnosed in all locales.

**Table 1:** **Detection of antigens of** ***Dirofilaria immitis*** ^**a**^ **in canine blood samples from different areas of Brazil**

**Region/locales**	**Antigens of** ***D. immitis***	
	**Positive/total**	**%**
**South**		
Florianópolis, SC	3/146	2.1
Araquari, SC	11/150	7.3
Guaratuba, PR	12/49	24.5
Guaraqueçaba, PR	7/22	31.8
Pontal do Paraná, PR	31/118	26.3
Combined	64/485	13.2
**Southeast**		
Guarujá, SP	4/142	2.8
Bertioga, SP	7/92	7.6
Mangaratiba, RJ	23/141	16.3
Niterói, RJ	92/157	58.6
Cabo Frio, RJ	11/40	27.5
Armação de Búzios, RJ	23/37	62.2
Combined	160/609	26.3
**Northeast**		
Lauro de Freitas, BA	30/148	20.3
Salvador, BA	24/120	20.0
Recife, PE	22/60	36.7
Itamaracá, PE	54/109	49.5
Combined	130/437	29.7
**Overall Total**	**354/1531**	**23.1**

^a^Tested with Witness® HW (heartworm) antigen test kit, Zoetis, USA.

The lowest area rate (13.2%) was found in the southern states, which have a milder climate. Nonetheless, even in the southern area, in less urbanized locales with better preserved natural resources, some states had rates higher than the overall prevalence in the survey (Table 1, Figure 1). Anthropization and climate seem to have had the greatest influence in the southeast, in which 26.3% of dogs tested positive. In Guarujá, a locale with mild temperatures (10.5° to 34.5°C) and which is highly impacted by industrial activities, 2.8% of the samples tested positive. In Niterói, where nature is better preserved, despite being located in a metropolitan area and temperature range from 10.1° to 41°C, 58.6% of the samples tested positive; and at Armação de Búzios, a well-preserved summer resort, 62.2% of samples tested positive (Table 1, Figure 2). The collective rate of dogs positive for heartworm in the southeast area was 26.3%. The overall regional rate of positive tests in the northeast was 29.7%, with the two biggest cities of the region (Salvador and Recife) displaying high rates (20% and 36.7%, respectively) in areas that are less anthropized (Table 1, Figure 3).

### Results in areas with low prevalence

In areas where a low prevalence of heartworm infection was detected (<20%), owner perception of the density of mosquitoes and the majority of individual canine characteristics were not associated with positive test results (Table 2). Two individual characteristics that could be associated with positive results were gray coat color versus other coat colors (χ^2^ = 17.93; df = 4; *P* = 0.001) and size (weight). Small dogs (<5 kg), medium-large dogs, and large dogs (>25 kg) were less associated with positive test results than were medium-small dogs (5–15 kg) (χ^2^ = 19.37; df = 3; *P* < 0.001) (Table 2).

**Table 2:** **Number and percentage positive for antigens of** ***Dirofilaria immitis*** ^**a**^ **in canine blood samples according to the relative prevalence in areas sampled**

	***D. immitis*** **antigens**			
**Categories**	**Areas with <20% prevalence**		**Areas with ≥20% prevalence**	
	**Positive/total**	**%**	**Positive/total**	**%**
Hair coat length				
Short	35/430	8.1	217/555	39.1^†^
Medium	9/185	4.9	77/252	30.6*
Long	4/56	7.1	12/53	22.6*
Predominant hair color				
White	19/226	8.4*^†^	35/159	22.0*
Black	13/205	6.3^†^	116/305	38.0^†^
Golden	9/150	6.0^†^	109/260	41.9^†^
Brown	3/75	4.0^†^	37/103	35.9^†^
Gray	4/15	26.7*	9/33	27.3^†^*
Life style				
Outdoors	38/583	6.5	200/509	39.3*
Indoors	10/88	11.4	106/351	30.2^†^
Age (yr)				
1–2	23/330	7.0	57/204	27.9*
>2–4	8/111	7.2	95/268	35.5^†^
>4–6	9/126	7.1	71/174	40.8^†^
>6	8/104	7.7	83/214	38.89^†^
Length of time at locale (yr)^b^				
1–2	15/205	7.3	78/245	31.8
>2–5	23/266	8.6	135/364	37.1
>5	10/200	5.0	93/250	37.2
Travel^b^				
No	45/650	6.9	296/813	36.4
Yes	3/20	15.0	10/47	21.3
Weight (kg)				
<5	9/185	4.9^†^	33/126	26.2^†^
5–15 kg	33/266	12.4*	145/457	31.7^†^
>15–25 kg	2/116	1.7^†^	83/194	42.8*
>25 kg	4/104	3.9^†^	45/83	54.2*
Hemoparasites^c^				
Never	44/638	6.9	245/704	34.8
Yes (previously)	1/18	5.6	33/70	47.1
Received doxycycline^d^				
No	47/636	7.4	243/706	34.4
Yes	1/32	3.1	31/79	39.2

^a^Witness® HW (heartworm) antigen test kit, Zoetis, USA; ^b^Data not provided for one animal; ^c^Data not provided for 101 animals; ^d^Data not provided for 78 animals. Different symbols (* or ^†^) within columns indicate significant difference (*P* < 0.05).

### Results in areas with high prevalence

At locales where positive test results were high (≥20%), owner perception of mosquito density also showed no association with canine heartworm infection (*χ*^*2*^ = 1.23; df = 2; *P* = 0.540). However, hair coat length, predominant coat color, life style, age, and size all demonstrated an effect on prevalence of heartworm. Short-haired dogs were positive for heartworm antigen more often than dogs with medium coat length (χ^2^ = 5.46; df = 1; *P =* 0.027) or dogs with long hair (χ^2^ = 5.58; df = 1; *P* = 0.024). Dogs with white hair coats tested negative for heartworm antigen more often than dogs with black (χ^2^ = 11.5; df = 1; *P* < 0.001), golden (χ^2^ = 17.34; df = 1; *P* < 0.001), or brown coats (χ^2^ = 5.391; df = 1; *P* = 0.020). Outdoor animals showed higher probability of testing positive than those kept indoors most of the time (χ^2^ = 7.103; df = 1; *P* < 0.008). Younger dogs (1–2 years) were less likely to test positive than were dogs of other (older) age groups (χ^2^ = 8.23; df = 3; *P* = 0.042). The percentage of large dogs (>25 kg) that tested positive was statistically similar to that for medium-large dogs (15–25 kg) (χ^2^ = 2.61; df = 1; *P* = 0.106), and dogs in both of these two size (weight) groups tested positive significantly more often than medium-small or small dogs (χ^2^ = 19.46; df = 1; *P* < 0.001) (Table 2). Medium-small dogs and small dogs had similar percentages of positive tests (χ^2^ = 1.18; df = 1; *P* = 0.277).

### Clinical signs and physical categories

Physical variables that had a significant influence on test results included age group (χ^2^ = 31.1; df = 3; *P* < 0.001); size (χ^2^ = 21.3; df = 3; *P* < 0.001), and breed (χ^2^ = 22.7; df = 2; *P* < 0.001) (Table 3).

**Table 3:** **Percentage of positive canine tests for heartworm antigens** ^**a**^ **within physical category and according to clinical signs**

**Category**				**Coughing**		**Syncope**		**Dyspnea**		**Exercise intolerance**		**Weight loss**	
	**No. Pos**	**Total**	**%**	**No. Pos**	**No. Neg**	**No. Pos**	**No. Neg**	**No. Pos**	**No. Neg**	**No. Pos**	**No. Neg**	**No. Pos**	**No. Neg**
**Age** **(yr)**													
1–2	80	534	15.0*	6 (7.5)	21 (4.6)	1 (1.3)	1 (0.2)	2 (2.5)	7 (1.5)	3 (3.8)	20 (4.4)	5 (6.3)	22 (4.8)
>2–4	103	379	27.2^†^	11 (10.7)	36 (13.0)	0 (0.0)	2 (0.7)	3 (2.9)	7 (2.5)	9 (8.7)	22 (8.0)	8 (7.8)	26 (9.4)
>4–6	80	300	26.7^†^	13 (16.3)	23 (10.6)	0 (0.0)	0 (0.0)	6 (7.5)	7 (3.2)	7 (8.8)	23 (10.6)	8 (10.0)	13 (6.0)
>6	91	318	28.6^†^	20 (22.0)	48 (21.1)	3 (3.3)	9 (4.0)	4 (4.4)	15 (6.6)	17 (18.7)	48 (21.1)	12 (13.2)	40 (17.6)
**Weight** **(kg)**													
<5	42	311	13.5*	8 (19.0)	35 (13.0)	0 (0.0)	3 (1.1)	1 (2.4)	9 (3.3)	6 (14.3)	22 (8.2)	9 (21.4)	23 (8.6)
5–15	178	723	24.6^†^	24 (13.5)	70 (12.8)	2 (1.1)	7 (1.3)	7 (3.9)	20 (3.7)	16 (9.0)	55 (10.1)	9 (5.1)	39 (7.2)
>15–25	85	310	27.4^†^	9 (10.6)	17 (7.6)	0 (0.0)	1 (0.4)	2 (2.4)	4 (1.8)	7 (8.2)	22 (9.8)	8 (9.4)	21 (9.3)
>25	49	187	26.2^†^	9 (18.4)	6 (4.3)	2 (4.1)	1 (0.7)	5 (10.2)	3 (2.2)	7 (14.3)	14 (10.1)	7 (14.3)	18 (13.0)
**Breed**													
Mongrel	245	924	26.5^†^	31 (12.7)	67 (9.9)	3 (1.2)	8 (1.2)	5 (2.0)	18 (2.7)	19 (7.6)	56 (8.3)	18 (7.3)	54 (8.0)
Mixed	45	330	13.6*	11 (24.4)	23 (8.1)	0 (0.0)	0 (0.0)	3 (6.7)	6 (2.1)	5 (11.1)	22 (7.7)	5 (11.1)	20 (7.0)
Purebred	64	277	23.1^†^	8 (12.5)	37 (17.4)	1 (1.6)	4 (1.9)	7 (10.9)	11 (5.2)	12 (18.8)	34 (16.0)	10 (15.6)	27 (12.7)
**Sex**													
Female	196	838	23.4	21 (10.7)	56 (8.7)	1 (0.5)	4 (0.6)	4 (2.0)	18 (2.8)	18 (9.2)	59 (9.2)	15 (7.7)	46 (7.2)
Male	158	693	22.8	29 (18.4)	72 (13.5)	3 (1.9)	8 (1.5)	11 (7.0)	18 (3.4)	18 (11.4)	54 (10.1)	18 (11.4)	55 (10.3)
**Data missing**				3	9	2	5	4	11	3	7	1	10

^a^Tested with Witness® HW (heartworm) antigen test kit, Zoetis, USA. Different symbols (* or ^†^) within a column indicate difference among category variables (*P* < 0.01).

Overall, presence of clinical signs reported by owners was not predictive of positive tests results for *D. immitis* (Table 3). However, when associating clinical signs with age or size, without regard for the test result, some correlation could be detected (Table 3): Age was correlated with frequency of coughing (χ^2^ = 52.2; df = 3; *P* < 0.001), exercise intolerance (χ^2^ = 60.5; df = 3; *P* < 0.001), and weight loss (χ^2^ = 33.3; df = 3; *P* < 0.001). Size of the animals was predictive of the presence of coughing (χ^2^ = 8.33; df = 3; *P* = 0.040) and weight loss (χ^2^ = 10.1; df = 3; *P* = 0.018); however, it was not predictive of exercise intolerance.

## Discussion

The presence of *D. immitis* was detected at all locales in the survey, demonstrating that the reduction, or in some areas, the supposed disappearance of heartworm observed by small animal practitioners early in the new millennium [13] has been replaced by an observed increase in the presence of the parasite in these areas of Brazil. We found the overall percentage of positive *D. immitis* antigen test results, despite differences in diagnostic methodologies and inclusion criteria, was higher in all three Brazilian regions compared with findings in previous studies and when the pooled prevalence (3.9%) of the three surveyed regions observed previously [9] is compared with the current result (23.1%), it is irrefutable that there is a recrudescence of heartworm in these areas.

Notwithstanding all locales being coastal, differences in landscape due to human action seem to have influenced test results in some areas. Guarujá (state of São Paulo) is a locale where, in the past, 14.2% of examined dogs were positive for heartworm infection [2] and in the current study, prevalence of heartworm was only 2.8%. Also, in Florianópolis (state of Santa Catarina), prevalence decreased from 12% reported in 1992 [4] to 2.1% in the current study. Both cities are structured. Guarujá is affected by industrial pollution from neighboring cities, and Florianópolis experienced a human population boom during the past decades. Although human densification enhances the canine population, which in turn facilitates the transmission of heartworm [18], it transforms the environment, usually making it inhospitable to the majority of mosquito-vector species [19,20], thereby disturbing transmission.

On the other hand, at conserved estuaries, such as in the lowlands of Paraná, where the local economy is based on tourism or artisanal fishing, even though their annual mean temperatures generally range from 14° to 22°C [21], the prevalence of heartworm-infected dogs was higher than that observed in the more urbanized cities of Salvador or Lauro de Freitas, where the average temperature is much warmer (20°–28°C) [21]. Therefore, if on one hand, temperature is directly related to the number of mosquito generations produced [22-24] and with the speed of parasite development in the mosquito vectors [25], the level of environmental conservation seems to play a crucial role in maintaining dense mosquito populations.

The perception of dog owners regarding the presence of mosquitoes was not associated with the prevalence of heartworm detected by testing for *D. immitis* antigen with a reliable commercial antigen test kit. This lack of perception suggests that inhabitants in these areas are accustomed to the presence of vectors and, therefore, are unwilling to control mosquitoes, which likely contribute to enhancing *D. immitis* transmission.

In areas where the prevalence of infected dogs was lower than 20%, a higher percentage of dogs with gray hair coat tested positive; however, the sample size for this hair color was small (15/1531) and may have biased the results. At areas with a prevalence 20% or higher, the majority of dogs with a white hair coat tested negative for heartworm. The prevalence of heartworm infection in these dogs with a white hair coat was significantly lower than among dogs with black, golden, or brown hair color. If mosquitoes can perceive different colors, as suggested by Maranhão [26], it is possible that the white hair coat may play a protective role in areas where heartworm transmission challenge is high. In addition to hair coat color, the length of the hair coat (long), and life style (living primarily indoors) appeared to be associated with reduced heartworm infections, presumably because these characteristics interfere with the vectors’ ability to locate hosts and obtain blood meals [27]. Considering that short hair coat presumably provides mosquitoes with better access to a dog’s skin [28-30] and that dogs that stay primarily outdoors are exposed to the sylvatic and the more efficient mosquito vectors that are hemi-synanthropic and exophylic [29,30], these characteristics also may have played a key role in increasing the percentage of positive test results.

Despite these findings of statistical association of certain physical characteristics, these results suggest that the relationship of canine individual characteristics (hair coat color or length, and outdoors life style) to test results is minor, mainly because it could only be detected where challenge was high. In previously surveyed areas, where the prevalence of heartworm infection was lower (10.4% and 15%), interference by these factors could not be detected [27,31].

The fact that the length of time the dogs lived at enzootic locales did not increase the percentage of positive test results suggests that infections occur soon after the animal is introduced to a location. Also, traveling experiences did not influence the results, suggesting that dogs in the areas surveyed most likely became infected in the home region; however, it would be expected that these animals could eventually spread the infection if and when they traveled abroad, as suggested before [32].

The prevalence of heartworm infection among younger dogs (1–2 years) may have been smaller than for the older age groups due to the long prepatent period of the infection (6–7 months) [14], especially because the time dogs lived at the enzootic areas did not demonstrate an effect on test results. An association between the small size (i.e. dogs weighing <5 or 5–15 kg) and indoor life style may have been the reason for smaller animals to have fewer positive test results as compared with the prevalence among larger dogs (i.e. dogs weighing 15–25 or >25 kg), as observed previously [33,34].

Breeds have been previously compared as purebred versus mongrels, with most reports showing no difference among different breed categories [35,36], although one report showed purebred dogs to have more positive test results [37]. Therefore, it is difficult to interpret the lower percentage of positive test results in mixed-breed dogs compared with results in mongrels or purebred dogs, and the current results may have occurred by chance alone.

Independent of *D. immitis* antigen test results, testing of various characteristic variables against the reported clinical signs reported by owners indicated that older dogs presented a higher prevalence of exercise intolerance, cough, and weight loss than younger dogs. With regard to size, smaller dogs were more inclined to have a cough and lose weight more frequently, possibly due to heart or respiratory diseases associated with other etiologies [38,39].

## Conclusions

Canine *D. immitis* infection was detected in every region surveyed, with a tendency to have higher percentage of positive test results where nature is better conserved. In areas where heartworm was highly prevalent, there were significantly more cases of *D. immitis* infection in large dogs, outdoor dogs, and dogs with short hair coats. Clinical signs observed by owners did not correlate with positive test results, suggesting that clinical signs of heartworm infections are rare or, at best, subtle, and call for other methods to be used for detection of heartworm infection, particularly for light or early infections. Therefore, small animal practitioners must include heartworm routine screening tests in every Brazilian dog’s annual evaluation protocol and make sure to have uninfected dogs on prevention.

## References

[1] Guerrero J, Vezzoni A, Ducos-de-Lahitte, Bussieras J, Rojo FA, Ortega LM, Rodenas A, Bulman GM, Larson MH, Labarthe NV, Charles T, Bordin EL, Otto GF (1989). Distribution of *Dirofilaria immitis* in selected areas of Europe and South America. Proceedings of Heartworm Symposium'89.

[2] Duque-Araujo AM, Labarthe NV, Luvisário SL, Reina D: **Filariose canina no Estado de São Paulo-Brasil.** In *Proceedings of IV Congresso Ibérico de Parasitologia 1995 Santiago de Compostela, Espanha.* Edited by Congresso Ibérico de Parasitologia. Congresso Ibérico de Parasitologia; 1995:93–95

[3] Labarthe NV, Almosny NR, Guerrero J, Duque-Araujo AM (1997). Description of the occurrence of canine dirofilariasis in the State of Rio de Janeiro, Brazil. Mem Inst Oswaldo Cruz.

[4] Labarthe NV, Araujo AM, Bordin EL, Larsson ME, Guerrero J (1992). Update on the distribution of *Dirofilaria immitis* in dogs in Brazil. Proceedings of the WSAVA World Congress '92.

[5] Reifur L, Soccol VT, Montiani-Ferreira F (2001). Prevalence of filariosis in dogs from the coast of Parana State Brasil: Emphasizing *Dirofilaria immitis*. Proceedings of the 10th Triennial Heartworm Symposium.

[6] Alves LC, Cole EF, Athayde ACR (1993). Prevalência da filariose canina no Bairro de Dois Irmãos. Rev Bras Parasitol Vet.

[7] Pimentel A, Alves LC: **Estudos epidemiológicos preliminares na população canina de Itamaraca-PE [abstract].** *Simpósio Nacional de Filariose*. 1987

[8] Barros MTG, Santos EP, Gondim LFP, Almeida MAO (1991). Frequencia de microfilarias de *Dirofilaria immitis* (Leidy,1856) em caes dos municipios de Salvador e Lauro de Freitas. Rev bras saude prod anim.

[9] Labarthe N, de Campos PM, Barbarini O, McKee W, Coimbra CA, Hoskins J (2003). Serologic prevalence of *Dirofilaria immitis, Ehrlichia cani*s, and *Borrelia burgdorferi* infections in Brazil. Vet Ther.

[10] Labarthe N, Guerrero J (2005). Epidemiology of heartworm: what is happening in South America and Mexico?. Vet Parasitol.

[11] Bandi C, Trees AJ, Brattig NW (2001). Wolbachia in filarial nematodes: evolutionary aspects and implications for the pathogenesis and treatment of filarial diseases. Vet Parasitol.

[12] Bendas AJR, Paiva JP, Doria MI, Mendes-de-Almeida F, Branco AS, Silvano DRB, Valle LG, Labarthe NV (2007). Ocorrência de *Dirofilaria immitis* no entorno de um case diagnosticado na Zona Sul do rio de Janeiro/RJ, Brasil. Acta Sci Vet.

[13] Labarthe NV (2009). As filarias estão de volta. Prepare-se. Nosso Clinico.

[14] Knight DH, King LG (2004). Heartworm infection. Textbook of Respiratory Disease in Dogs and Cats.

[15] Grandi G, Zivicnjak R, Beck R, Genchi C, Rinaldi L, Cringoli G (2014). Pathogenesis of *Dirofilaria* spp. infections. Mappe parassitologiche-Dirofilaria: Dirofilaria immitis and D. repens in dog and cat and human infections.

[16] Venco L, Genchi C, Rinaldi L, Cringoli G (2007). Heartworm (Dirofilaria immitis) disease in dogs. Mappe parassitologiche-Dirofilaria: Dirofilaria immitis and D. repens in dog and cat and human infections.

[17] **IBGE** [http://censo2010.ibge.gov.br]. 2010.

[18] Walters LL, Soll MD, Knight DH (1995). Rick factors for heartworm infection in northern California. Proceedings of the Heartworm Symposium '95.

[19] Paula MB, Gomes AC (2007). Culicidae (Diptera) in a dam construction area in the state of São Paulo, Brazil. Rev Saúde Públ.

[20] Teodoro U, Guilherma ALF, Lazaovei AL, La Salvia FV, Fukushigue Y, Spinosa R, Ferreira AMEMC, Lima EM (1995). Culicídeos do lago de Itaipu, no rio Paraná, sul do Brasil. Rev Saúde Públ.

[21] **Cactáceas Brasileira**s. [http://www.brcactaceae.org/p_index.html]. 2014.

[22] Calado DC, Silva MAN (2002). Avaliação da influencia da temperatura sobre o desenvolvimento de *Aedes albopicturs*. Rev Saúde Públ.

[23] Cônsoli RAGB, Lourenço-de-Oliveira R (1994). Principais mosquitos de importância sanitária no Brasil.

[24] Roiz D, Ruiz S, Soriguer R, Figuerola J (2014). Climatic effects on mosquito abundance in Mediterranean wetlands. Parasit Vectors.

[25] McTier TL, McCall JW, Supakorndej N, Soll MD, Knight DH (1995). Features of adult heartworm antigen test kits. Proceedings of the Heartworm Symposium '95.

[26] Maranhão ZC (1976). Entomologia Geral.

[27] Almeida MAO, Barros MTG, Santos EP, Ayres MCC, Guimaraes JE, Gondim LFP (2001). Parasitismo de cães por microfilárias de *Dirofilaria immitis*: influência da raça, sexo e idade. Rev Bras Aaude Prod Anim.

[28] Forantinni OP, Gomes AC, Natal D, Kakitani I, Marucci D (1987). Frequência domiciliar e endofilia de mosquitos Culicidae no Vale do Ribeira, São Paulo, Brasil. Rev Saúde Públ.

[29] Forantinni OP, Gomes AC, Natal D, Kakitani I, Marucci D (1987). Preferências alimentares de mosquitos Culicidae no Vale do Ribeira, São Paulo, Brasil. Rev Saúde Públ.

[30] Forantinni OP, Kakitani I, Massad EM, Marucci D (1995). Studies on mosquitoes (Diptera: Culicidae) and anthropic environment. 9-Synathropyc and epidemiological vector roles of *Aedes scapularis* in south-eastern Brazil. Rev Saúde Públ.

[31] Araujo RT, Marcondes CB, Bastos LC, Sartor DC (2003). Canine dirofilariasis in the region of Conceicao Lagoon, Florianopolis, and in the Military Police kennel, Sao Jose, State of Santa Catarina, Brazil. Vet Parasitol.

[32] Rinaldi L, Musella V, Marzatico G, Mortarino M, Genchi C, Cringoli G (2014). Mapping and modeling *Dirofilaria* infections in Europe. Parasit Vectors.

[33] Ortega-Mora LM, Gomez-Bautista M, Rojo-Vazqueza F, Roidenasb A, Guerrero J (1991). A survey of the prevalence of canine filariasis in Spain. Prev Vet Med.

[34] Theis JH, Franti C, Lambert L, Giammattei V, Parker V, Lee G (1984). Risk factors for heartworm infection in dogs living in Sierra Nevada foothills and Sacramento Valley counties; public health implications. California Vet.

[35] Rajamanickam C, Wiesenhutter E, Zin FM, Hamid J (1985). The incidence of canine haematozoa in Peninsular Malaysia. Vet Parasitol.

[36] Souza SSHVC (1992). Diagnostico da dirofilariose através da detecção de antigenos circulantes em cães do estado do Rio de Janeiro. MSc thesis.

[37] Kan SP, Rajah KV, Dissanaike AS (1977). Survey of dirofilariasis among dogs in Serembam, Malaysia. Vet Parasitol.

[38] Gompf RE, Goodwin JK, Tilley LP (2002). A historia e o exame fisico. Manual de cardiologia para câes e gatos.

[39] Kittleson MD, Kittleson MD, Kienle RD (1998). Signal, history and physical examination. Small animal cardiovascular medicine.

